# Higher intraoperative arterial-to-end-tidal carbon dioxide gradients are associated with postoperative respiratory morbidity after lung cancer resection: a retrospective INSPIRE cohort study

**DOI:** 10.3389/fmed.2026.1995794

**Published:** 2026-10-15

**Authors:** Long Li, Dan Wang, Qingyi Zhou, Bin Shang

**Affiliations:** 1 The Second Affiliated Hospital of Bengbu Medical University, Bengbu, China; 2 Department of Cardio-Thoracic Surgery, The Second Affiliated Hospital of Bengbu Medical University, Bengbu, China; 3 Department of Cardiology, The Second Affiliated Hospital of Bengbu Medical University, Bengbu, China

**Keywords:** arterial blood gas, capnography, carbon dioxide, lung cancer, postoperative respiratory morbidity, pulmonary resection

## Abstract

**Background:**

The arterial-to-end-tidal carbon dioxide gradient may reflect impaired gas exchange during thoracic surgery. We examined its association with postoperative respiratory morbidity after lung cancer resection.

**Methods:**

We conducted a retrospective cohort study using INSPIRE version 1.4.2. Each intraoperative arterial carbon dioxide (PaCO_2_) measurement was paired with the nearest positive end-tidal carbon dioxide (EtCO_2_) measurement within 10 min, and the primary exposure was each patient's median gradient. Respiratory morbidity was defined from new respiratory diagnoses and qualifying postoperative ventilation records during the index admission. We used multivariable regression, repeated-measurement analyses, sensitivity analyses, and exploratory internally validated prediction models.

**Results:**

Among 2,665 eligible patients, 85 (3.2%) had respiratory morbidity. A valid gradient was available in 2,527 patients; the primary complete-case analysis included 2,436 patients and 80 events. Each 5 mmHg higher gradient was associated with greater morbidity (adjusted odds ratio 1.53, 95% confidence interval 1.17–2.00; adjusted risk ratio 1.48, 1.14–1.91). Standardised risk increased from 2.28% at the first gradient quartile to 3.46% at the third, a difference of 1.18 percentage points (0.49–1.83). In a *post hoc* comparison, adding the gradient to a model already containing PaCO_2_ and clinical covariates did not materially change internally cross-validated discrimination (AUROC 0.7324 vs. 0.7325).

**Conclusion:**

A higher intraoperative PaCO_2_–EtCO_2_ gradient was associated with postoperative respiratory morbidity after lung cancer resection, but its incremental prognostic value beyond PaCO_2_ was limited in exploratory analyses. Prospective external validation is required before clinical use.

## Introduction

Postoperative respiratory complications can delay recovery after lung cancer resection. Baseline respiratory impairment and operative characteristics contribute to pulmonary risk, although the performance of risk models varies across surgical populations (1, 2). A European cohort developed a separate prediction score for postoperative respiratory failure, reflecting the need to distinguish this outcome from broader pulmonary-complication composites (3). A recent systematic review found limited external validation and a high risk of bias in most postoperative pulmonary prediction models (4). Intraoperative measurements may help characterise respiratory risk that is not captured by preoperative assessment alone.

Ventilation during lung resection must maintain gas exchange while limiting adverse effects on lung mechanics and haemodynamics. An initial trial of driving-pressure-guided ventilation reported fewer pulmonary complications, whereas a larger multicentre trial improved respiratory mechanics without reducing its primary pulmonary outcome (5, 6). Trials have also examined lower tidal volumes with positive end-expiratory pressure and recruitment, and an individualised perioperative open-lung strategy (7, 8). In PROTHOR, higher positive end-expiratory pressure with recruitment did not significantly reduce postoperative pulmonary complications and was associated with more hypotension and arrhythmias (9). These results support studying gas exchange alongside ventilatory settings and haemodynamics.

End-tidal carbon dioxide (EtCO_2_) is routinely monitored during anaesthesia. Lower resting EtCO_2_ was associated with postoperative pulmonary complications in a prospective study of lung-resection candidates (10). Associations between intraoperative EtCO_2_ and postoperative outcomes have also been reported in a secondary analysis of two randomised ventilation trials and a retrospective thoracoscopic-lobectomy cohort (11, 12). Physiological studies in lung-resection candidates suggest that both arterial carbon dioxide tension (PaCO_2_) and the dead-space to tidal-volume ratio contribute to ventilatory inefficiency (13). Measuring the difference between arterial and end-tidal carbon dioxide may therefore provide information that EtCO_2_ alone cannot capture.

The PaCO_2_–EtCO_2_ gradient varies during thoracic anaesthesia, particularly during one-lung ventilation, when EtCO_2_ may be an unreliable estimate of PaCO_2_ (14, 15). A wider gradient may reflect ventilation-perfusion heterogeneity or changes in pulmonary perfusion, but it is not equivalent to physiological dead-space fraction measured by volumetric capnography (16). We investigated the association between the median intraoperative PaCO_2_-EtCO_2_ gradient and in-hospital postoperative respiratory morbidity after lung cancer resection. We also examined repeated measurements, absolute risk, and sensitivity to exposure and outcome definitions. Trajectory analyses and landmark prediction were exploratory objectives; detailed trajectory methods are provided in the Supplementary Material.

## Materials and methods

### Study design and data source

We conducted a retrospective cohort study using INSPIRE version 1.4.2, accessed through PhysioNet. This credentialed-access database contains perioperative data from Seoul National University Hospital for operations performed from January 2011 through December 2020 (17–19). It links surgical characteristics, diagnoses, laboratory results, intraoperative monitoring, and postoperative observations. For de-identification, numerical values are discretised and timestamps are aggregated to a maximum resolution of 5 min. Reported values therefore differ from the original bedside measurements, and matching timestamps do not necessarily indicate simultaneous sampling.

The original INSPIRE study was approved by the Institutional Review Board of Seoul National University Hospital (H-2210-078-1368), with a waiver of informed consent. Public release was approved separately following de-identification review (17, 18). We used the data under the applicable data-use requirements and made no attempt to re-identify patients. Reporting followed the Strengthening the Reporting of Observational Studies in Epidemiology statement (20).

### Study population

We screened operations performed under general anaesthesia in the cardiothoracic service with ICD-10-PCS prefixes beginning with 0BB (excision) or 0BT (resection). INSPIRE excludes patients younger than 18 years or older than 90 years before age categorisation; we applied no additional age exclusion to the released categories. Lung malignancy was defined by an ICD-10-CM C34 diagnosis recorded between 180 days before operation start and the operation date. For each patient, we selected the first eligible operation by start time, using the operation identifier to resolve ties. We then restricted the cohort to lung procedures, identified by C, D, F, G, H, J, K, L, or M in the fourth code position. For the principal analysis, five-character procedure prefixes were required to match the first five characters of codes in the December 1, 2021 release of the Centers for Medicare & Medicaid Services (CMS) 2022 ICD-10-PCS code set (21).

We included one operation per patient because postoperative observations are linked to patients without an operation identifier. Outcomes were assessed during the index admission. Patients who underwent a later operation during that admission were retained in the principal cohort and excluded in a sensitivity analysis. Figure 1 shows patient selection and the population used for the primary complete-case analysis.

**Figure 1:**
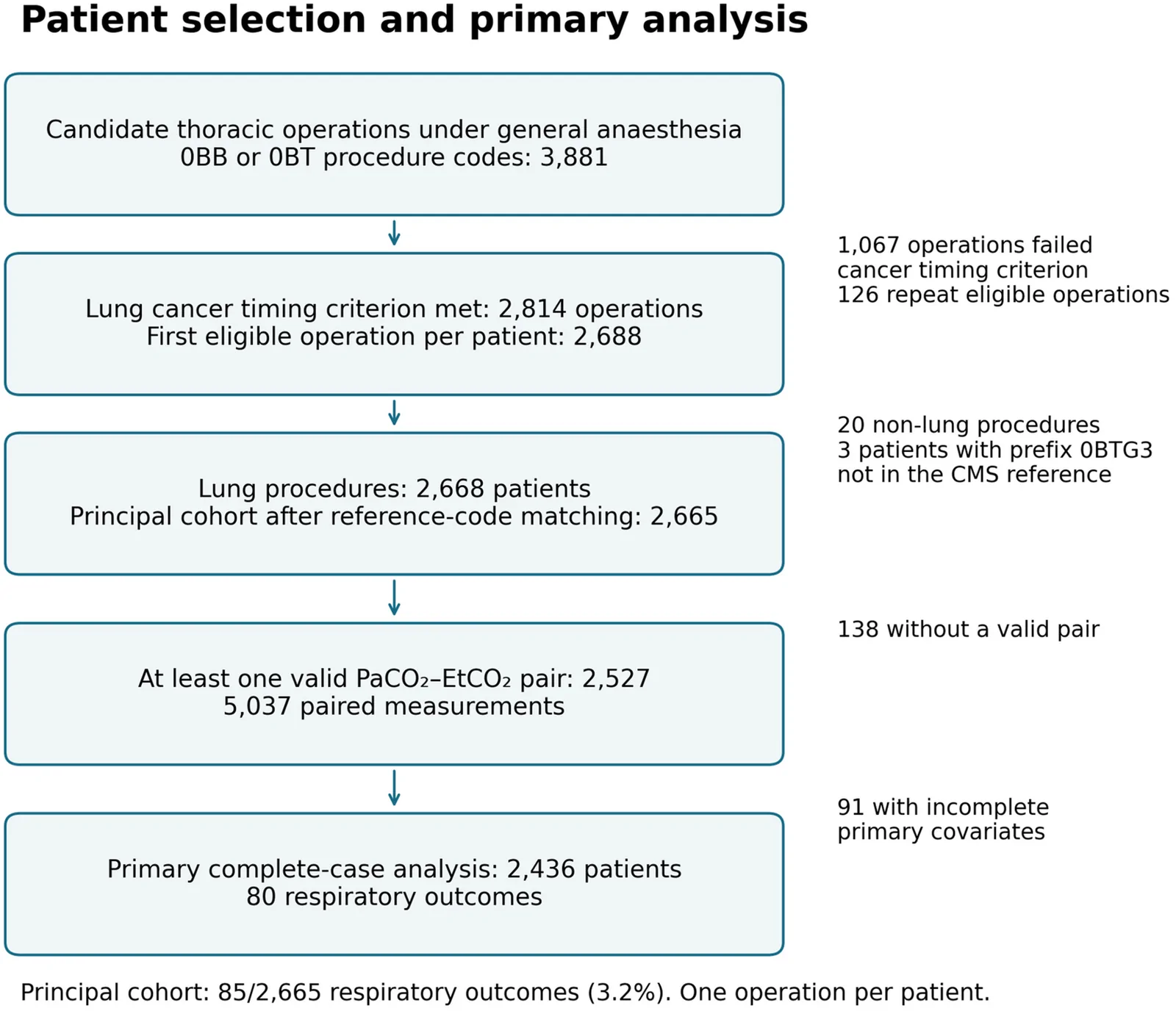
Patient selection. Operations were selected by lung-cancer diagnosis timing, first eligible operation per patient, lung body-part codes, and matching to the CMS 2022 ICD-10-PCS reference file. Three patients with procedure prefix 0BTG3 were excluded by the reference-matching criterion. The principal cohort contained 2,665 patients and 85 respiratory events; the primary complete-case analysis included 2,436 patients and 80 events. The three excluded patients were included in a sensitivity analysis, giving a broader cohort of 2,668 patients before complete-case restriction.

### Exposure definition and measurement processing

PaCO_2_ and EtCO_2_ measurements were restricted to the interval between recorded operation start and end. We removed exact duplicate records and used the median when different values shared the same operation, parameter, and timestamp. After excluding non-positive PaCO_2_ and EtCO_2_ values, each arterial measurement was paired with the nearest EtCO_2_ measurement within ±10 min. The earlier EtCO_2_ measurement was chosen for equidistant ties. This procedure allowed pairing with a nearby positive value when the nearest raw EtCO_2_ value was zero. A sensitivity analysis instead performed matching before excluding zero EtCO_2_ values, without rematching discarded pairs.

The gradient was calculated as PaCO_2_ minus EtCO_2_ in mmHg. The primary exposure was each patient's median of the paired gradients, modelled per 5 mmHg increase. No threshold was selected from the outcome data. Alternative summaries were the mean, first, last, and maximum gradient. For the normalised exposure, we calculated (PaCO_2_−EtCO_2_)/PaCO_2_ for each pair and used the patient median, modelled per 0.05 increase. Sensitivity analyses used identical timestamps or a ±5 minute pairing window, excluded negative gradients, or adjusted for the number of pairs. Negative gradients were retained in the primary analysis. We also described the number of pairs per patient and the time differences within pairs.

### Outcome definition

The primary outcome was in-hospital postoperative respiratory morbidity, defined from the database as either a new respiratory diagnosis or a qualifying ventilation record. A new diagnosis was a J18, J69, J80, J81, J95, or J96 code recorded from postoperative day 1 through index discharge, with no record of the same three-character code during the preceding 180 days through the operation date. Diagnosis windows were anchored to the calendar day of operation start. We excluded diagnoses recorded on the operation date because their timing relative to surgery was unknown. A qualifying ventilation record was a positive binary ventilation entry at least 6 h after operating-room exit and no later than index discharge.

The ventilation criterion indicated support recorded at or after 6 h; it did not establish 6 h of continuous ventilation. The data could not reliably distinguish planned from unplanned support or invasive from non-invasive ventilation. Patients without a qualifying diagnosis or ventilation record were classified as having no recorded event. To examine incomplete observation, a sensitivity analysis required documented ward observations during the admission. The available data did not permit clinical adjudication using consensus definitions of postoperative pulmonary complications (22). We therefore retained the term respiratory morbidity for this database-derived outcome. Diagnosis and ventilation components could overlap.

One sensitivity analysis retained new diagnoses throughout the index admission but restricted ventilation records to 6–72 h after operating-room exit. A separate early respiratory endpoint combined new diagnoses on postoperative calendar days 1–7 with ventilation recorded between 6 and 168 h after operating-room exit. Diagnosis windows used calendar days and ventilation windows used elapsed hours because the two sources had different time resolutions.

### Covariates and data cleaning

The primary model adjusted for age per 10 years, sex, body mass index (BMI), American Society of Anesthesiologists (ASA) physical status (I, II, or III or higher), emergency surgery, preoperative chronic obstructive pulmonary disease (COPD), procedure type, and surgical approach. COPD was defined by a J44 diagnosis within 180 days through the operation date. Age and BMI were entered as linear terms. Procedure type was coded as excision (0BB) or resection (0BT); the fifth code character identified open (0) or percutaneous endoscopic (4) approaches in the principal cohort. A sensitivity model replaced the binary procedure term with lung excision (0BB), lobar resection (0BT with fourth character C, D, F, G, H, or J), or whole-lung resection (0BT with K, L, or M). These categories approximated surgical extent from the recorded codes without anatomical adjudication. Operation duration accumulated over the same period as the exposure and was added in a separate model rather than the primary adjustment set.

Covariate selection was summarised in a *post hoc* conceptual directed acyclic graph (Supplementary Figure S1). The primary adjustment set comprised measured baseline and operative-context variables considered plausible common determinants of the gradient and postoperative respiratory risk: age, sex, BMI, ASA physical status, emergency status, COPD, procedure type, and surgical approach. Operation duration, MAP, VT/PBW, FiO_2_, and PEEP were examined separately because their temporal relation to a whole-operation gradient was not unambiguous. The DAG was developed during revision to clarify assumptions and does not claim causal identification.

Height values of 1,400–1,900 were treated as millimetres and divided by 10. Height outside 100–200 cm and weight outside 10–200 kg were treated as missing. BMI was calculated as weight in kilograms divided by height in metres squared. Predicted body weight (PBW) was derived from height and sex for tidal-volume standardisation, following the ARDS Network approach (23). We used the inch-based equations: 50 + 2.3 × (height in inches−60) kg for men and 45.5 + 2.3 × (height in inches−60) kg for women, with height converted from centimetres by dividing by 2.54.

Additional association models included operation duration with median intraoperative invasive mean arterial pressure (MAP), tidal volume divided by PBW (VT/PBW), and inspired oxygen fraction (FiO_2_); duration with preoperative albumin, haemoglobin, C-reactive protein, and creatinine; or preoperative PaCO_2_ and arterial oxygen tension (PaO_2_). Intraoperative summaries were medians of positive observations rather than time-weighted means. Inspired oxygen percentages were divided by 100 for the expanded association models. Landmark prediction used the released percentage scale before standardisation within training folds. Baseline laboratory values were the last measurements obtained within 7 days before anaesthesia start. Three records with anaesthesia start after operation start were assigned missing baseline laboratory values. The source compliance parameter was based on plateau pressure and was not interpreted as dynamic compliance.

### Primary statistical analysis

Continuous characteristics are reported as medians and interquartile ranges, and categorical characteristics as counts and percentages with observed denominators. Baseline differences were described using standardised mean differences (SMDs); hypothesis-testing *P* values were not used. The primary multivariable logistic model contained 10 slope parameters and an intercept. Modified Poisson regression with robust sandwich variance estimated risk ratios (24). Separate PaCO_2_, EtCO_2_, and gradient models used the same primary complete-case cohort and covariates for AIC comparison. To assess small-sample bias, the primary model was also refitted using Firth penalised likelihood with profile penalised-likelihood confidence intervals and a penalised likelihood-ratio test.

To assess incremental value beyond PaCO_2_, a *post hoc* nested analysis compared the primary covariates plus median PaCO_2_ with the same model additionally containing the median gradient. We compared AIC and used a one-degree-of-freedom likelihood-ratio test. Predictive performance was compared with repeated stratified 5-fold cross-validation (10 repeats; identical folds), using averaged out-of-fold probabilities and 2,000 paired patient bootstrap resamples for AUROC and Brier-score differences.

We estimated marginally standardised risks by averaging predicted risks over the primary complete-case cohort with the gradient set to its first quartile, median, or third quartile. Percentile confidence intervals for these risks, their difference, and their ratio were obtained from 1,000 patient bootstrap samples with model refitting. The three exposure values remained fixed at the original sample values. The continuous risk curve used the same reference population, with pointwise intervals from 1,000 multivariate-normal coefficient draws. A cubic B-spline with three exposure degrees of freedom was compared with the linear term using a two-degree-of-freedom likelihood-ratio test. We report two-sided *P* values and 95% confidence intervals. Secondary *P* values were nominal except for the Holm-adjusted interaction tests described below.

### Repeated measurements and secondary analyses

Each arterial–end-tidal pair was matched to MAP within ±10 min. We used Gaussian generalised estimating equations (GEE) with an exchangeable working correlation and robust variance to model the gradient by subsequent outcome group, contemporaneous MAP, centred normalised surgical progress, squared progress, and the primary covariates (25). An outcome-by-progress interaction tested whether the linear change differed between groups. A random-intercept model fitted by restricted maximum likelihood provided a second estimate of the group difference.

We also fitted a random-intercept measurement model containing MAP and linear and quadratic progress, without the outcome. For each patient, the empirical-Bayes gradient level was the fixed reference intercept plus the patient random intercept, evaluated at MAP 80 mmHg and 50% surgical progress. We then estimated its association with respiratory morbidity per 5 mmHg increase, adjusting for the primary covariates. A patient cluster bootstrap with 500 replicates refitted both models to account for uncertainty from both stages. These estimates summarise each patient's observed measurements under the model's reference conditions.

Respiratory categories were no event, diagnosis without qualifying ventilation, and qualifying ventilation with or without a diagnosis. We assessed the proportional-odds assumption using a stacked-threshold GEE Wald test of all threshold-by-slope interactions. The assumption was rejected, so we reported separate binary component analyses rather than interpreting a common OR or category-probability curves as evidence of increasing clinical severity.

We estimated Aalen-Johansen cumulative incidence by gradient quartile from operating-room exit, treating discharge or in-hospital death before respiratory morbidity as competing events (26). Respiratory events took precedence when timestamps were identical. Diagnosis dates indicated when a diagnosis was recorded, rather than adjudicated onset. Among patients discharged alive, adjusted quantile regression estimated differences in the 50th, 75th, and 90th percentiles of postoperative stay (27).

### Missing data and sensitivity analyses

The primary analysis included patients with complete covariates and an observed gradient. We used 20 chained-equation imputations for missing BMI and ASA among patients with a valid gradient; absent arterial gases were not imputed (28). Predictive mean matching used five donors, Gaussian parameter perturbation, 20 burn-in sweeps, and five sweeps between retained imputations. The imputation model included the outcome, gradient, primary covariates, and operation duration. ASA was imputed as an ordinal category score and entered categorically in the analysis. Estimates were combined using Rubin's rules.

For patients with complete primary baseline covariates, we modelled the probability of observing a gradient using those covariates and operation duration. Stabilised weights were calculated as the marginal observation probability divided by the patient's fitted probability and were not truncated. Weighted regression used empirical weighted-score sandwich variance conditional on the fitted observation model. This analysis addressed selection explained by the measured predictors, subject to correct specification of the observation model.

For a reviewer-requested PEEP sensitivity analysis, the nearest PEEP measurement within ±10 min of each valid PaCO_2_ sampling time was identified, with the earlier value selected for equidistant ties. The patient-level PEEP covariate was the median of these pair-aligned values. The expanded model containing the primary covariates, operation duration, MAP, VT/PBW, and FiO_2_ was refitted with and without pair-aligned PEEP in the same complete-case population. A secondary sensitivity analysis used each patient's median PEEP across the entire operation.

We evaluated 108 specifications combining four outcomes, three pairing windows (0, 5, and 10 min), three exposure summaries (median, mean, and normalised median), and three covariate sets. Additional sensitivity analyses addressed surgical extent, whole-lung resection, influential observations, the three unmatched procedure prefixes, and removal of individual diagnosis components. Separate models tested interactions with sex, COPD, and resection vs. excision. The 108 specifications were treated as a dependent robustness grid rather than independent confirmatory tests; no global multiplicity correction was applied and their confidence intervals are nominal. Holm adjustment was applied to the three interaction tests. An E-value assessed sensitivity to unmeasured confounding (29).

### Exploratory trajectory and prediction analyses

Detailed principal-component and hidden Markov model trajectory methods were moved to the Supplementary Material. These analyses were hypothesis-generating; HMM states were treated as data-derived statistical states rather than validated physiological or clinical phenotypes (Supplementary Methods and Supplementary Figure S2).

Landmark models included patients with a recorded operation duration of at least 30, 60, 90, or 120 min, irrespective of complete-case status. Baseline predictors were age, sex, BMI, ASA, emergency status, COPD, and preoperative albumin, haemoglobin, C-reactive protein, and creatinine. ASA was represented by indicators for I, II, III or higher, and missing. The Physiology model added six summaries for each of EtCO_2_, invasive MAP, VT/PBW, and inspired oxygen percentage: median, last value, standard deviation, interquartile range, linear slope per hour, and observation count. Summaries used observations from operation start through the landmark; slopes required at least two distinct timestamps. The Physiology + arterial gases model also included median available PaCO_2_, median available PaCO_2_–EtCO_2_ gradient, arterial-sample count, and valid-pair count. Pairing was repeated at each landmark using the ±10 min rule, with both measurements required on or before that time. Predictors excluded final procedure coding, final operation duration, gradients from the whole operation, and PCA/HMM features. Sampling timestamps were available, but laboratory reporting delays were unknown.

Ridge logistic models used nested stratified cross-validation with five outer folds, three inner folds, and three repeats. Numeric predictors were median-imputed with missingness indicators and standardised within training folds; preprocessing was repeated within the inner folds. Inner-validation log loss selected C from 0.01, 0.1, 1, or 10. Averaged out-of-fold probabilities were used for AUROC, AUPRC, Brier score, calibration-in-the-large, and calibration slope. Conditional intervals used 1,000 resamples of the fixed averaged predictions and did not include uncertainty from repeating model development. Reporting followed TRIPOD + AI principles (30). Validation was internal only; no external cohort was used.

### Analysis specification and software

The study was not preregistered and the analysis was reconstructed after data access. The median-gradient association with index-admission respiratory morbidity was the principal inferential analysis; secondary, sensitivity, specification, trajectory, and prediction analyses were interpreted as supportive or exploratory. Analyses used Python 3.12.14, pandas 2.2.3, NumPy 2.3.5, SciPy 1.17.0, statsmodels 0.15.0, scikit-learn 1.8.0, hmmlearn 0.3.3, and patsy 1.0.3. Figures were produced with matplotlib 3.10.8.

A base random seed S = 20260911 was used for primary bootstrap resampling and the subsequent risk-curve coefficient simulation. Multiple imputation used S + 1 and empirical-Bayes bootstrap resampling used S + 2. Prediction used S + r for outer folds and S + 100 + 5r + f for inner folds, with repeat *r* = 0, 1, or 2 and outer-fold *f* = 0, 1, 2, 3, or 4. At landmark t minutes, metric-interval resampling used S + t and paired model-comparison resampling used S + 500 + t.

## Results

### Study population and paired measurements

Of 3,881 candidate operations, 2,814 met the lung-cancer diagnosis timing criterion. Selecting the first eligible operation per patient left 2,688 patients, of whom 20 had non-lung procedures. Three further patients had procedure prefix 0BTG3, which did not match the CMS reference file. The principal cohort therefore comprised 2,665 patients (Figure 1). Respiratory morbidity occurred in 85 patients (3.2%): 64 had a new respiratory diagnosis and 28 had a qualifying ventilation record, including seven with both. J18 was recorded in 36 patients, J69 in two, J80 in five, J95 in 26, and J96 in four; no new J81 diagnosis was recorded. Table 1 summarises the principal cohort.

**Table 1:** Characteristics of the principal cohort by respiratory outcome.

Characteristic	No outcome (*n* = 2,580)	Outcome (*n* = 85)	Missing (n)	SMD
Age, years	65.0 (55.0–70.0)	70.0 (65.0–75.0)	0	0.55
Male sex	1,388/2,580 (53.8%)	73/85 (85.9%)	0	0.74
BMI, kg/m^2^	23.4 (21.5–25.7)	23.7 (20.6–25.7)	23	0.10
ASA physical status: I	518/2,499 (20.7%)	7/81 (8.6%)	85	0.35
ASA physical status: II	1,807/2,499 (72.3%)	58/81 (71.6%)	85	0.02
ASA physical status: III or higher	174/2,499 (7.0%)	16/81 (19.8%)	85	0.38
Emergency surgery	85/2,580 (3.3%)	1/85 (1.2%)	0	0.14
Preoperative COPD	216/2,580 (8.4%)	15/85 (17.6%)	0	0.28
Procedure extent proxy: Lobar resection	1,255/2,580 (48.6%)	45/85 (52.9%)	0	0.09
Procedure extent proxy: Lung excision	1,179/2,580 (45.7%)	34/85 (40.0%)	0	0.11
Procedure extent proxy: Whole-lung resection	146/2,580 (5.7%)	6/85 (7.1%)	0	0.06
Open approach	100/2,580 (3.9%)	7/85 (8.2%)	0	0.18
Operation duration, min	130.0 (105.0–170.0)	185.0 (135.0–235.0)	0	0.64
Median gradient, mmHg	9.8 (7.1–12.4)	12.4 (9.7–16.0)	138	0.64
PaCO_2_, mmHg	44.7 (41.1–48.2)	47.0 (43.6–51.0)	138	0.42
Paired EtCO_2_, mmHg	35.0 (32.5–37.0)	35.0 (32.5–37.0)	138	0.08
Preoperative albumin, g/dL	4.1 (4.0–4.3)	4.0 (3.8–4.2)	66	0.24
Preoperative haemoglobin, g/dL	13.2 (12.2–14.2)	13.2 (12.5–14.2)	42	0.02
Preoperative CRP, mg/dL	0.1 (0.0–0.2)	0.2 (0.1–0.8)	194	0.23
Preoperative creatinine, mg/dL	0.8 (0.7–0.9)	0.9 (0.8–1.0)	37	0.43
Postoperative stay, days	4.6 (3.5–6.5)	10.5 (7.3–20.3)	0	0.86
In-hospital death	1/2,580 (0.0%)	7/85 (8.2%)	0	0.42

Continuous values are medians (interquartile ranges); categorical values are n/observed denominator (%). Missing counts refer to all 2,665 patients in the principal cohort and are repeated for levels of the same categorical variable. Standardised mean differences were calculated using observed values and were used descriptively to characterise between-group imbalance; no hypothesis-testing *P* values were used for baseline comparisons. Surgical extent was inferred from procedure codes. Outcomes and postoperative stay are presented descriptively and were not baseline adjustment variables. BMI, body mass index; ASA, American Society of Anesthesiologists physical status; COPD, chronic obstructive pulmonary disease; CRP, C-reactive protein; SMD, standardised mean difference.

A valid gradient was available in 2,527 patients (94.8%), providing 5,037 pairs; 4,975 (98.8%) shared a timestamp and 5,021 (99.7%) were within 5 min. The median was two pairs (IQR 1–2): 1,109 patients (43.9%) contributed one, 824 (32.6%) two, 300 (11.9%) three, 256 (10.1%) four or five, and 38 (1.5%) six or more. The primary complete-case analysis included 2,436 patients and 80 events. Median gradient was 12.4 mmHg (9.7–15.4) in patients with respiratory morbidity and 9.8 mmHg (7.0–12.4) in those without it (Figure 2B). In the full principal cohort, BMI, ASA, and gradient were missing in 23, 85, and 138 patients, respectively.

**Figure 2:**
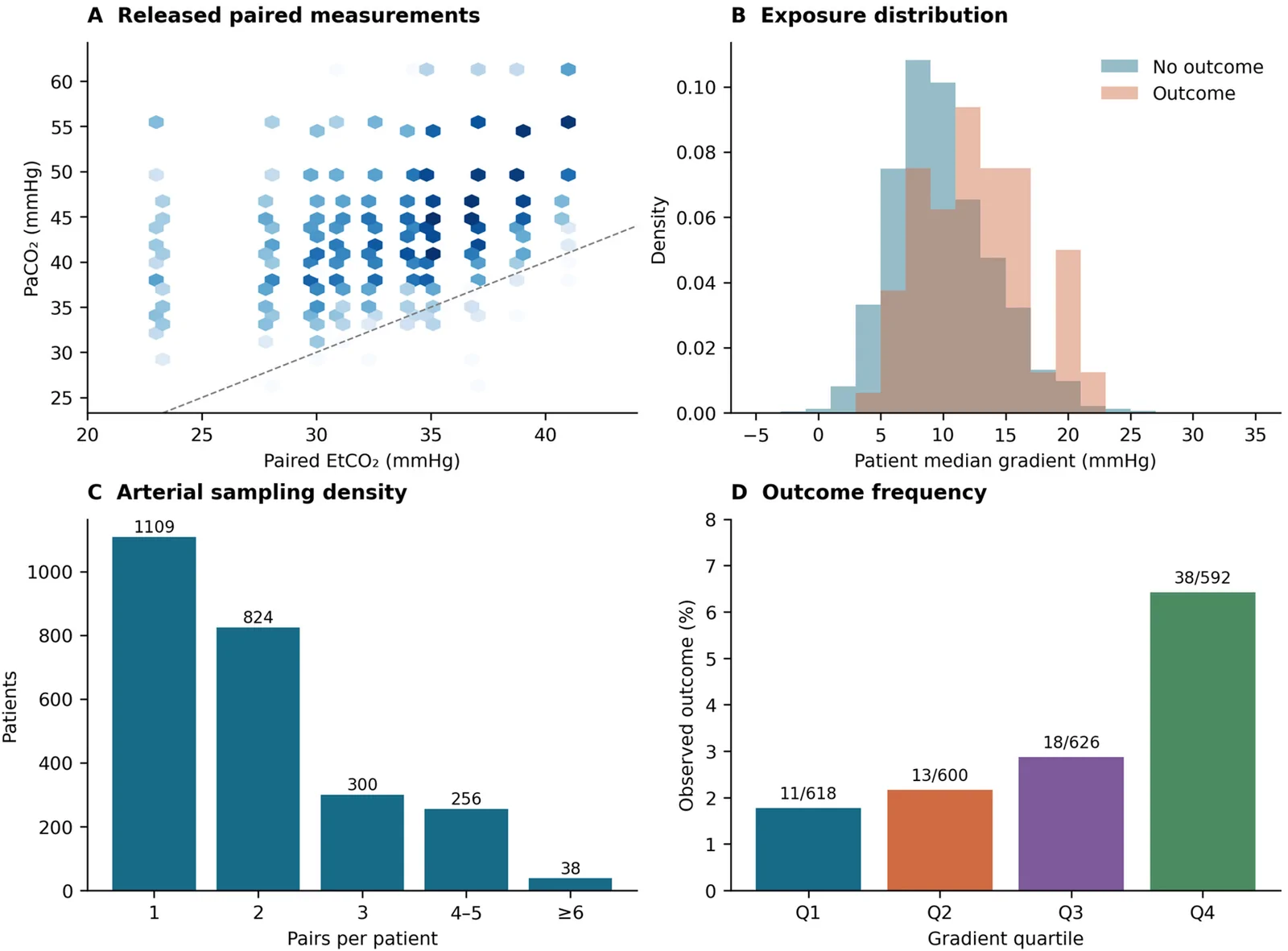
Paired measurements and respiratory morbidity. **(A)** PaCO_2_ and EtCO_2_ measurements from 2,527 patients in the principal cohort (5,037 pairs). The dashed line denotes equality; colours indicate logarithmic pair density. **(B)** Distribution of patient median gradients in the primary complete-case cohort (2,436 patients; 80 events). **(C)** Pair counts among the 2,527 patients with a gradient. **(D)** Respiratory morbidity throughout the index admission by gradient quartile in the primary complete-case cohort, with event counts and denominators. Tied gradient values produce unequal quartile sizes. Numerical values and timestamps have undergone privacy transformations, and measurements from the same patient are correlated.

### Primary association and absolute risk

Each 5 mmHg higher median gradient was associated with an adjusted OR of 1.53 (95% CI 1.17–2.00; *P* = 0.002). Modified Poisson regression estimated a risk ratio of 1.48 (1.14–1.91; *P* = 0.003). Associations remained positive in the expanded adjustment models (Table 2; Figure 3A). The spline model did not improve fit over the linear exposure term (nonlinearity *P* = 0.553). Firth penalised logistic regression using the same 2,436 patients and 80 events yielded a closely similar OR of 1.52 per 5 mmHg higher gradient (95% profile penalised-likelihood CI 1.16–1.98; *P* = 0.002).

**Table 2:** Primary, expanded, and sensitivity association analyses.

Analysis	Patients/events	Effect (95% CI)	*P*
Primary logistic model	2,436/80	1.53 (1.17–2.00)	0.002
Firth penalised logistic model	2,436/80	1.52 (1.16–1.98)	0.002
Modified Poisson risk ratio	2,436/80	1.48 (1.14–1.91)	0.003
Add operation duration	2,436/80	1.60 (1.22–2.09)	<0.001
Add duration, MAP, VT/PBW and FiO_2_	2,276/76	1.67 (1.24–2.24)	<0.001
Add duration, MAP, VT/PBW, FiO_2_, and pair-aligned PEEP	2,137/72	1.68 (1.23–2.29)	0.001
Add duration and preoperative laboratory values	2,263/76	1.61 (1.21–2.14)	<0.001
Add preoperative PaCO_2_ and PaO_2_	1,933/60	1.68 (1.21–2.33)	0.002
Twenty-imputation analysis	2,527/85	1.57 (1.21–2.03)	<0.001
Observation weighting	2,436/80	1.52 (1.15–2.02)	0.003
Ventilation limited to 6–72 h	2,436/74	1.51 (1.14–1.99)	0.004
Mean gradient	2,436/80	1.53 (1.17–2.01)	0.002
First gradient	2,436/80	1.39 (1.09–1.78)	0.008
Last gradient	2,436/80	1.29 (1.01–1.65)	0.039
Maximum gradient	2,436/80	1.45 (1.15–1.82)	0.002
Normalised gradient, per 0.05	2,436/80	1.26 (1.07–1.48)	0.005
Exact timestamp pairing	2,425/79	1.55 (1.18–2.03)	0.002
Pairing within ±5 min	2,434/80	1.52 (1.16–1.98)	0.002
Exclude negative paired gradients	2,432/80	1.53 (1.17–2.00)	0.002
Adjust number of pairs	2,436/80	1.56 (1.19–2.04)	0.001
Match before zero-EtCO_2_ exclusion	2,434/80	1.54 (1.18–2.02)	0.002
Require documented ward observations	2,423/79	1.51 (1.15–1.98)	0.003
Exclude later operation in index admission	2,413/73	1.49 (1.13–1.98)	0.005
Exclude whole-lung resection	2,300/74	1.58 (1.19–2.09)	0.001
Three-level surgical extent	2,436/80	1.53 (1.17–2.00)	0.002
Include three patients with prefix 0BTG3	2,439/80	1.53 (1.17–2.00)	0.002
Remove largest Cook distances	2,411/55	1.46 (1.05–2.02)	0.025

Effects are adjusted ORs per 5 mmHg higher gradient unless otherwise indicated. The modified Poisson model reports a risk ratio; the normalised-gradient effect is per 0.05. The Firth row reports a profile penalised-likelihood confidence interval and penalised likelihood-ratio *P* value. Primary covariates were age, sex, BMI, categorical ASA, emergency surgery, COPD, resection versus excision, and open versus endoscopic approach. The pair-aligned PEEP model additionally included operation duration, MAP, VT/PBW, FiO_2_, and median pair-aligned PEEP. When the three patients with prefix 0BTG3 were included, the approach indicator compared open with non-open procedures. Preoperative laboratory variables were albumin, haemoglobin, CRP, and creatinine. Unless specified, the exposure was the median gradient and the outcome was respiratory morbidity throughout the index admission. The 6–72 h analysis restricted ventilation to that window but retained diagnoses throughout the admission. The three-category extent model used lung excision, lobar resection, and whole-lung resection. Each row gives its analysis population. Observation-weighting variance was conditional on the fitted observation model. Removing the largest Cook distances excluded 25 outcome cases and served as an influence analysis.

**Figure 3:**
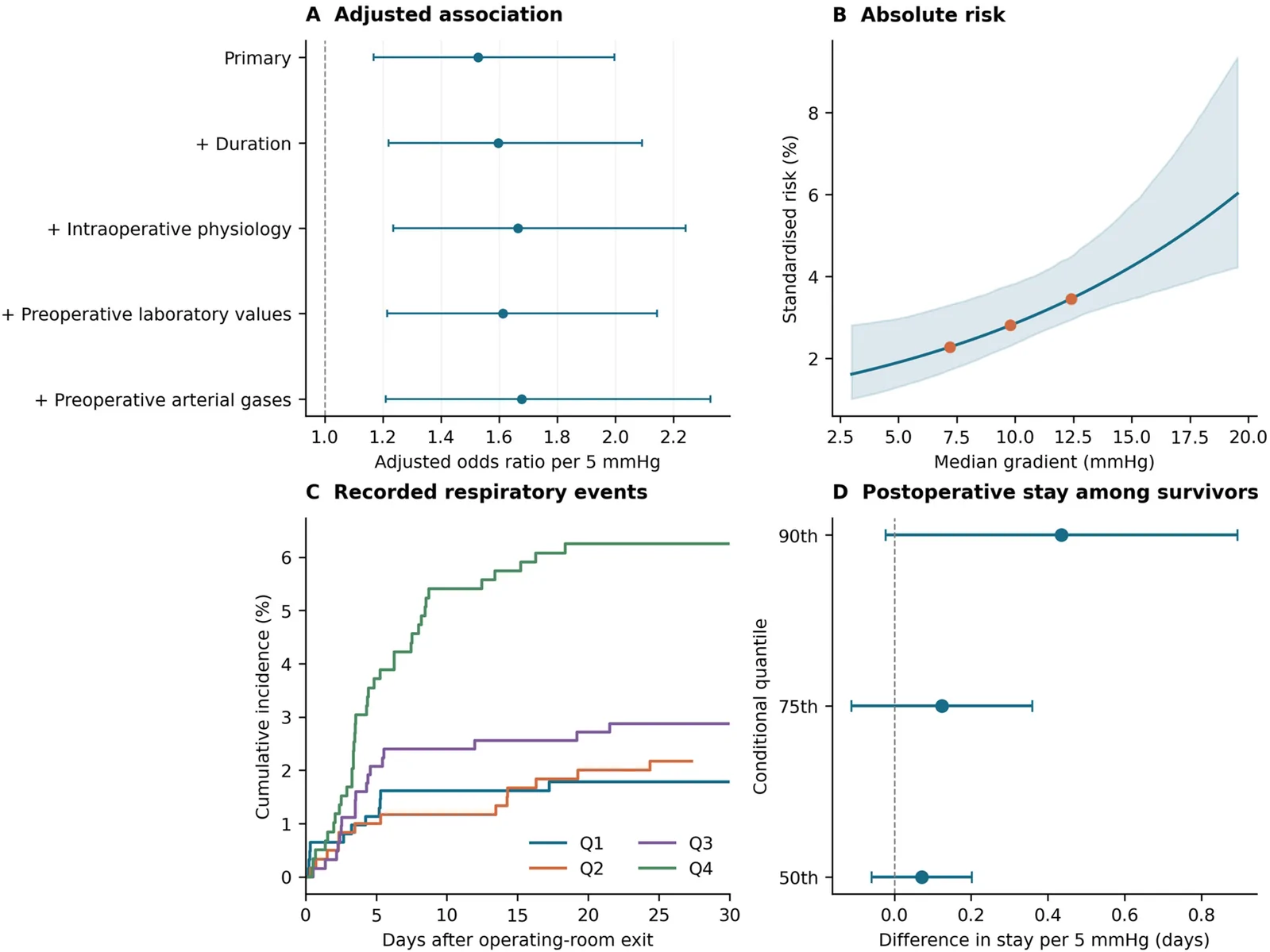
Gradient associations and postoperative outcomes. **(A)** Adjusted ORs with 95% confidence intervals for the primary model and selected expanded models; additional reviewer-requested sensitivity analyses are reported in Table 2. **(B)** Marginally standardised risk from the primary logistic model. The band shows pointwise intervals from coefficient simulation; dots mark the original gradient quartiles. Bootstrap intervals for the three reference risks and their contrast are reported in the text. **(C)** Aalen–Johansen cumulative incidence through 30 days by gradient quartile in the primary complete-case cohort, with discharge or death before an event treated as competing events. This 30-day incidence differs from the full-admission proportions in Figure 2D. **(D)** Adjusted quantile-regression estimates for postoperative stay among 2,429 survivors in the primary complete-case cohort, with 95% confidence intervals. Effects in A and D are per 5 mmHg higher median gradient.

Standardised risk was 2.28% (95% CI 1.55%–3.06%) at a gradient of 7.2 mmHg, 2.81% (2.15%–3.48%) at 9.8 mmHg, and 3.46% (2.74%–4.21%) at 12.4 mmHg. Comparing the third with the first gradient quartile gave a risk difference of 1.18 percentage points (0.49–1.83) and a standardised risk ratio of 1.52 (1.17–2.05; Figure 3B). All 1,000 bootstrap fits succeeded; the bootstrap interval for the primary OR was 1.18–2.06.

In the same 2,436 patients, AIC values were 646.95 for the gradient, 648.79 for PaCO_2_, and 655.78 for EtCO_2_. PaCO_2_ was associated with morbidity (OR 1.35 per 5 mmHg, 95% CI 1.09–1.68), whereas the EtCO_2_ interval included the null (OR 1.10, 0.77–1.56). In the *post hoc* nested analysis, adding the gradient to the PaCO_2_ model changed AIC from 648.79 to 648.57 (likelihood-ratio *P* = 0.136); the conditional gradient OR was 1.38 (0.91–2.10; *P* = 0.126). Cross-validated AUROC was 0.7324 with PaCO_2_ and 0.7325 after adding the gradient (difference 0.0001, 95% CI −0.0088 to 0.0093), and Brier scores were 0.03098 and 0.03106 (difference 0.00008, 95% CI −0.00008 to 0.00025).

### Repeated measurements and postoperative course

Repeated-measurement analyses included 4,849 pairs from the 2,436 patients in the primary analysis. The adjusted gradient difference between outcome groups was 1.42 mmHg (95% CI 0.44–2.41; *P* = 0.005) in the GEE model and 1.43 mmHg (0.59–2.28) in the random-intercept model. The outcome-by-progress interaction was −0.59 mmHg per full unit of relative progress (−2.38 to 1.21; *P* = 0.521). The measurement-model intraclass correlation was 0.513. Higher empirical-Bayes gradient levels were associated with respiratory morbidity (OR 1.92 per 5 mmHg, 95% CI 1.25–3.20). This interval accounted for refitting both model stages; all 500 bootstrap fits succeeded (Table 3).

**Table 3:** Repeated-measurement and respiratory-component analyses.

Analysis	Analysis population	Estimate (95% CI)	Scale
GEE outcome-group gradient difference	4,849 pairs/2,436 patients	1.42 (0.44–2.41)	mmHg
Random-intercept group difference	4,849 pairs/2,436 patients	1.43 (0.59–2.28)	mmHg
Empirical-Bayes adjusted gradient level	4,849 pairs/2,436 patients	1.92 (1.25–3.20)	OR per 5 mmHg
New respiratory diagnosis	2,436 patients/63 events	1.49 (1.11–2.02)	OR per 5 mmHg
Qualifying ventilation record	2,436 patients/23 events	1.84 (1.17–2.92)	OR per 5 mmHg
Composite excluding J18	2,436 patients/51 events	1.56 (1.12–2.16)	OR per 5 mmHg
Composite excluding J69	2,436 patients/80 events	1.53 (1.17–2.00)	OR per 5 mmHg
Composite excluding J80	2,436 patients/79 events	1.51 (1.15–1.98)	OR per 5 mmHg
Composite excluding J81	2,436 patients/80 events	1.53 (1.17–2.00)	OR per 5 mmHg
Composite excluding J95	2,436 patients/59 events	1.68 (1.24–2.27)	OR per 5 mmHg
Composite excluding J96	2,436 patients/80 events	1.53 (1.17–2.00)	OR per 5 mmHg

GEE, generalised estimating equations. The empirical-Bayes confidence interval was obtained from 500 patient bootstrap samples with both model stages refitted; the measurement-model intraclass correlation was 0.513. Diagnosis and ventilation components overlap. For each component-exclusion analysis, the listed diagnosis was removed while all other diagnoses and the ventilation criterion were retained. Overlap between components can leave the event count unchanged. The proportional-odds assumption was rejected (global Wald *χ*^2^ = 42.82, df = 10, *P* < 0.001); no common OR is therefore reported as an estimate of clinical severity.

The primary complete-case cohort included 2,356 patients without an event, 57 with a diagnosis but no qualifying ventilation, and 23 with qualifying ventilation. The proportional-odds assumption was rejected (Wald *χ*^2^ = 42.82, 10 degrees of freedom, *P* < 0.001), precluding interpretation of a common OR across these categories. Separate diagnosis and ventilation models gave ORs of 1.49 (95% CI 1.11–2.02) and 1.84 (1.17–2.92), respectively.

At 30 days, the cumulative incidence of recorded in-hospital respiratory morbidity was 6.25% in the highest gradient quartile, compared with 1.78%, 2.17%, and 2.88% in quartiles 1–3 (Figure 3C). Among 2,429 patients discharged alive, adjusted quantile-regression estimates per 5 mmHg higher gradient were 0.07 additional days at the median (95% CI −0.06 to 0.20), 0.12 at the 75th percentile (−0.11 to 0.36), and 0.44 at the 90th percentile (−0.02 to 0.89). All intervals included zero (Figure 3D).

### Sensitivity analyses

Multiple imputation gave an OR of 1.57 (95% CI 1.21–2.03) in 2,527 patients/85 events, and observation weighting gave 1.52 (1.15–2.02). E-values were 2.31 for the risk-ratio estimate and 1.55 for the confidence limit nearest the null. Pair-aligned PEEP was available in 2,256 patients (89.3%; median 5 cmH_2_O, IQR 4–5; range 0–8). In the same 2,137 patients/72 events, the gradient OR was 1.67 (1.22–2.27) without PEEP and 1.68 (1.23–2.29) with pair-aligned PEEP. Operation-median PEEP gave a similar estimate (OR 1.69, 1.24–2.30; 2,140 patients/72 events).

Restricting ventilation ascertainment to 6–72 h while retaining diagnoses throughout the admission yielded 74 events and an OR of 1.51 (95% CI 1.14–1.99) in the primary complete-case cohort. Matching before excluding zero EtCO_2_ values gave an OR of 1.54 (1.18–2.02) among 2,434 patients and 80 events. Including the three patients with procedure prefix 0BTG3 increased the complete-case population to 2,439 patients, with 80 events and an OR of 1.53 (1.17–2.00). Table 2 presents the alternative exposure, cohort, and surgical-extent analyses.

Across the 108 specifications, all point estimates were greater than 1 (Figure 4), but the models used overlapping populations and were treated as descriptive robustness analyses rather than independent tests. ORs for the first, last, and maximum gradient were 1.39 (95% CI 1.09–1.78), 1.29 (1.01–1.65), and 1.45 (1.15–1.82). Excluding the 25 observations with the largest Cook distances gave an OR of 1.46 (1.05–2.02). Associations remained positive after removing each diagnosis component (Table 3). Interaction ratios were 0.74 (0.37–1.47), 0.69 (0.35–1.38), and 0.64 (0.38–1.09) for sex, COPD, and surgical extent; Holm-adjusted *P* values were 0.596, 0.596, and 0.294, respectively.

**Figure 4:**
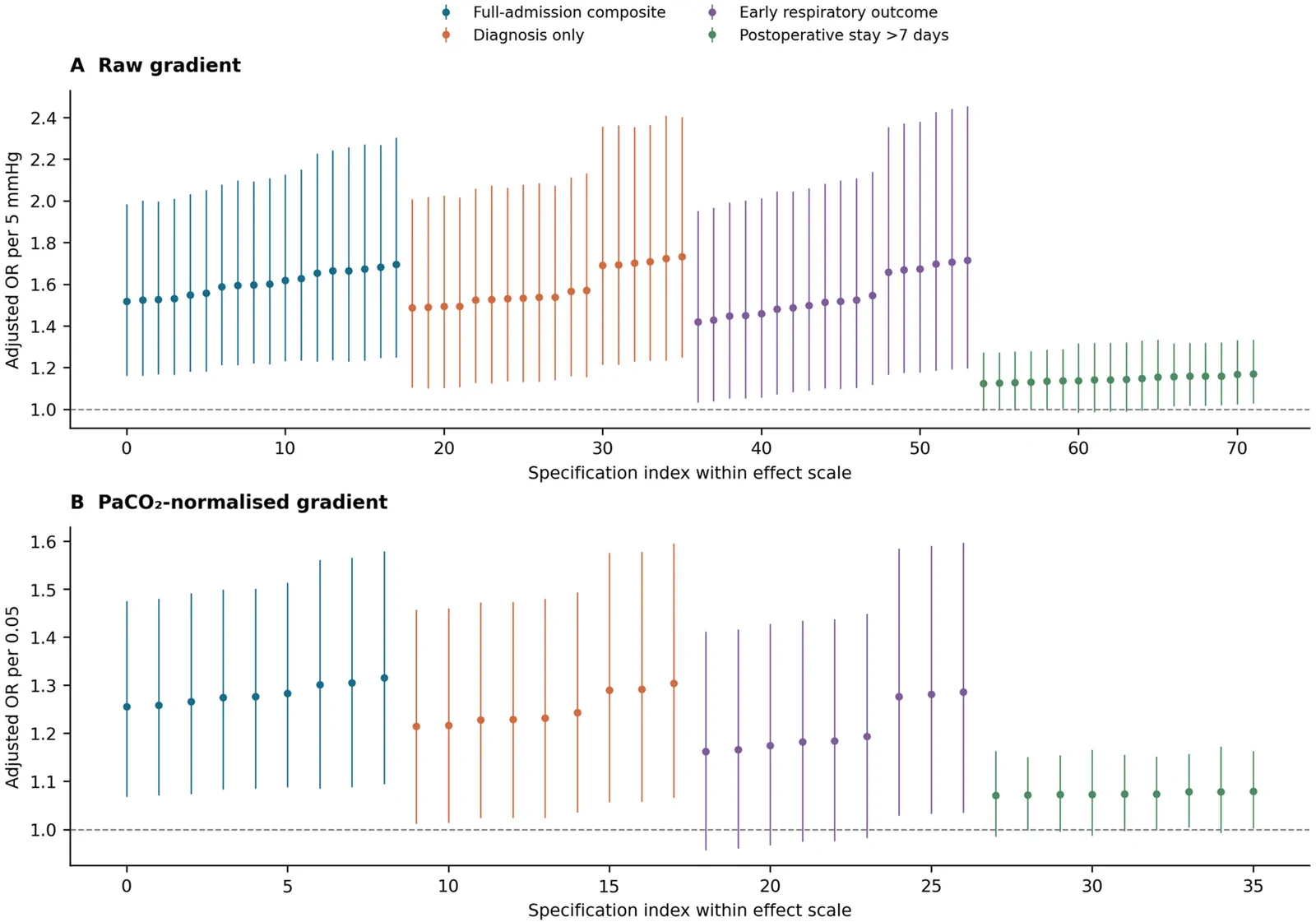
Associations across model specifications. **(A)** Seventy-two models of raw median or mean gradient, with effects per 5 mmHg. **(B)** Thirty-six models of the median pairwise PaCO_2_-normalised gradient, with effects per 0.05. Points show adjusted ORs with nominal 95% confidence intervals, ordered by estimate within outcome type. Models combine different pairing windows and covariate sets. Colours distinguish the full-admission composite, new diagnoses only, the early respiratory endpoint, and postoperative stay over 7 days. Postoperative stay is a recovery outcome. Confidence intervals are nominal and describe variation across analytical specifications; they are not multiplicity-adjusted simultaneous confidence intervals. The models use overlapping patients and do not represent independent replications.

### Exploratory trajectory and internally validated prediction analyses

Hypothesis-generating PCA and HMM trajectory analyses did not identify consistent additional associations beyond the patient-level gradient. Detailed methods, results, and the former main-text trajectory figure are provided in the Supplementary Material (Supplementary Figure S2).

The 30-, 60-, 90-, and 120 min landmark cohorts included 2,661, 2,579, 2,312, and 1,703 patients, with 85, 85, 83, and 72 events. At 90 min, AUROC was numerically highest for Physiology + arterial gases (0.734), vs. 0.718 for Physiology and 0.695 for Baseline; the paired-bootstrap difference vs. Baseline was 0.039 (95% CI −0.004 to 0.082). Corresponding AUPRC values were 0.103, 0.085, and 0.073, and Brier scores 0.0336, 0.0339, and 0.0342. Table 4 and Figure 5 use the same averaged out-of-fold probabilities. These analyses were internally validated only.

**Table 4:** Exploratory internally validated landmark prediction performance.

Time (min)	Model	N/events	AUROC (95% CI)	AUPRC (95% CI)	Brier score (95% CI)	CITL	Calibration slope
30	Baseline	2,661/85	0.700 (0.644–0.757)	0.068 (0.050–0.105)	0.0305 (0.0247–0.0362)	−0.0022	0.995
30	Physiology	2,661/85	0.689 (0.634–0.742)	0.065 (0.048–0.101)	0.0306 (0.0248–0.0363)	−0.0017	1.061
30	Physiology + arterial gases	2,661/85	0.701 (0.642–0.753)	0.073 (0.054–0.120)	0.0304 (0.0248–0.0362)	−0.0022	1.067
60	Baseline	2,579/85	0.692 (0.634–0.747)	0.065 (0.049–0.092)	0.0315 (0.0257–0.0385)	0.0034	0.943
60	Physiology	2,579/85	0.688 (0.634–0.741)	0.068 (0.051–0.104)	0.0315 (0.0256–0.0384)	−0.0017	1.027
60	Physiology + arterial gases	2,579/85	0.700 (0.643–0.753)	0.075 (0.055–0.116)	0.0313 (0.0255–0.0383)	−0.0023	1.013
90	Baseline	2,312/83	0.695 (0.634–0.749)	0.073 (0.052–0.107)	0.0342 (0.0273–0.0409)	−0.0011	0.895
90	Physiology	2,312/83	0.718 (0.661–0.771)	0.085 (0.060–0.130)	0.0339 (0.0270–0.0405)	0.0006	1.177
90	Physiology + arterial gases	2,312/83	0.734 (0.675–0.786)	0.103 (0.069–0.165)	0.0336 (0.0267–0.0401)	0.0017	1.172
120	Baseline	1,703/72	0.679 (0.622–0.738)	0.071 (0.053–0.098)	0.0401 (0.0318–0.0486)	−0.0037	1.195
120	Physiology	1,703/72	0.692 (0.635–0.749)	0.087 (0.062–0.141)	0.0398 (0.0317–0.0482)	−0.0042	1.169
120	Physiology + arterial gases	1,703/72	0.711 (0.656–0.766)	0.100 (0.067–0.170)	0.0396 (0.0315–0.0478)	−0.0036	1.143

AUROC, AUPRC, and Brier score are shown with conditional 95% percentile intervals from 1,000 patient bootstrap resamples of fixed out-of-fold probabilities. Probabilities were averaged across three repeats of nested cross-validation with five outer and three inner folds. The intervals do not include uncertainty from repeating model development. AUROC, area under the receiver operating characteristic curve; AUPRC, average precision. Brier score is the mean squared probability error. Calibration-in-the-large (CITL) is the intercept estimated with the prediction logit as an offset (ideal 0); calibration slope is estimated separately with a free intercept (ideal 1). Baseline uses preoperative predictors. Physiology adds EtCO_2_, MAP, VT/PBW, and inspired oxygen summaries; Physiology + arterial gases adds available PaCO_2_, gradient, and sampling counts. Sample sizes vary with landmark eligibility. All estimates are from internal validation within the INSPIRE cohort; no external validation cohort was used.

**Figure 5:**
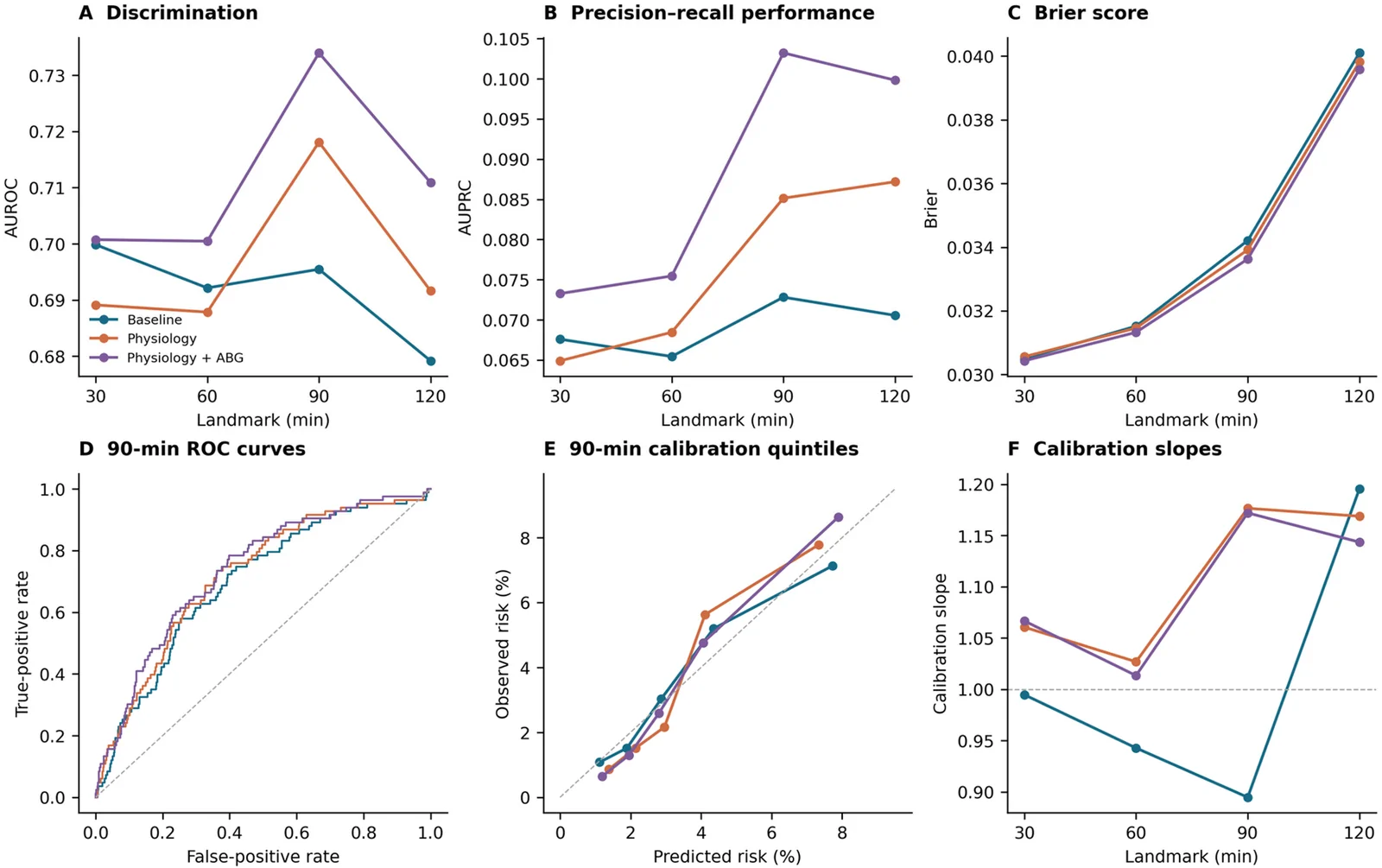
Exploratory landmark prediction. All panels use the same averaged out-of-fold probabilities. **(A–C)** AUROC, average precision (AUPRC), and Brier score at 30, 60, 90, and 120 min. **(D)** ROC curves at 90 min. **(E)** Observed and predicted risk by quintile at 90 min. **(F)** Calibration slope at each landmark. Baseline uses preoperative predictors; Physiology adds intraoperative physiological summaries; Physiology +ABG also includes available arterial-gas features (ABG, arterial blood gas). Probabilities were averaged over three repeats of five outer folds, with three inner folds for penalty selection. Preprocessing used training data only. Sample sizes and outcome prevalence vary with landmark eligibility; Table 4 provides corresponding denominators and performance measures. Validation was internal to the INSPIRE cohort; no external cohort or clinical implementation was evaluated.

## Discussion

Among 2,665 patients undergoing lung cancer resection, a higher median intraoperative PaCO_2_–EtCO_2_ gradient was associated with database-derived postoperative respiratory morbidity after adjustment for clinical and procedural factors. Each 5 mmHg increase corresponded to an adjusted OR of 1.53 and a risk ratio of 1.48, and standardised risk increased by 1.18 percentage points between the first and third gradient quartiles. The association was robust to several measured-data sensitivity analyses, but these findings establish an observational association rather than clinical predictive utility.

PaCO_2_ itself was associated with respiratory morbidity, and the gradient model had only a small AIC advantage over the PaCO_2_ model. More directly, the *post hoc* nested analysis showed that adding the gradient to PaCO_2_ and the primary clinical covariates produced only a 0.23-unit reduction in AIC, no clear likelihood-ratio improvement, and essentially no change in internally cross-validated AUROC or Brier score. The gradient coefficient was also attenuated when conditioned on PaCO_2_. Thus, the present data support an association with respiratory morbidity but do not demonstrate clinically meaningful incremental risk prediction beyond PaCO_2_.

The association has several plausible physiological explanations. PaCO_2_ reflects carbon dioxide production relative to effective alveolar ventilation, whereas EtCO_2_ is also influenced by regional perfusion, the sequence of lung emptying, and gas sampling. Studies of thoracic anaesthesia have documented variation in the arterial-end-tidal difference as operative conditions change (31, 32), and the accuracy of carbon-dioxide monitoring depends on measurement technique (33). EtCO_2_ has also been shown to track changes in cardiac output during altered haemodynamics (34). Adjustment for systemic MAP cannot fully account for these effects because cardiac output and pulmonary blood flow were not measured.

The gradient should be distinguished from physiological dead-space fraction. The Bohr equation uses alveolar and mixed-expired carbon dioxide, whereas the Enghoff modification substitutes arterial carbon dioxide and is also affected by shunt and ventilation-perfusion inequality (16). Neither calculation is equivalent to PaCO_2_ minus EtCO_2_. Although dead-space fraction predicts mortality in acute respiratory distress syndrome (35), that finding cannot be directly applied to our exposure. Studies of ventilatory inefficiency in lung-resection candidates support the relevance of PaCO_2_ and dead space to gas exchange (13), but direct dead-space measurements would be needed to determine their contribution to the present association.

Ventilation strategy may influence both the gradient and postoperative outcomes. Driving pressure was associated with pulmonary complications in an observational cohort of one-lung ventilation (36), while randomised trials of individualised ventilation have reported differing pulmonary outcomes (6, 8, 9). A trial of permissive hypercarbia improved intraoperative oxygenation without demonstrating fewer postoperative pulmonary complications (37). In our data, adjustment for pair-aligned PEEP changed the gradient OR only from 1.67 to 1.68 in the same analysis population, suggesting that measured PEEP alone did not explain the association. A recent prospective study using simultaneous arterial blood gas sampling and capnography in mechanically ventilated ICU patients also associated a larger PaCO_2_–EtCO_2_ gradient with 28-day mortality (38). The different setting, sampling protocol, and outcome make that study physiological context rather than external validation of our lung-resection findings.

Recent work places perioperative risk assessment after lung resection within a broader multimodal framework. A 2026 systematic review found that selected preoperative inflammation-based biomarkers may be associated with postoperative pulmonary morbidity, although evidence was heterogeneous and predominantly retrospective (39). A separate prolonged-air-leak review proposed integrating molecular biomarkers, clinical characteristics, and technology-derived signals in a conceptual precision-perioperative strategy (40). The gradient may represent a complementary intraoperative physiological signal, but our study did not test combined biomarker-physiology models.

Arterial sampling was sparse and non-standardised. Among patients with an observed gradient, 43.9% contributed only one pair and 76.5% contributed no more than two. The timing and duration of one-lung ventilation were unavailable, so individual pairs could not be assigned reliably to pre-, during-, or post-OLV phases. Repeated-measurement models appropriately accounted for clustering of available observations but provided limited information about individual temporal trajectories or phase-specific changes. The similar estimate after adjusting for pair count does not resolve this limitation.

The 3.2% event rate should not be interpreted as the incidence of the full spectrum of consensus-defined postoperative pulmonary complications. The database-derived endpoint did not systematically capture complications such as atelectasis or bronchospasm, diagnosis codes did not provide adjudicated onset or severity, and ventilation records could not distinguish planned from unplanned or invasive from non-invasive support. Both false-negative and false-positive classification are therefore possible. Associations were directionally consistent for diagnosis-only and ventilation-only components, when ventilation was restricted to 6–72 h, when ward observation was required, and when individual diagnosis categories were removed, but these analyses cannot validate the clinical accuracy of the endpoint.

The exploratory PCA and HMM analyses are now presented in the Supplementary Material as hypothesis-generating analyses; their HMM states are data-derived statistical constructs rather than validated phenotypes. Landmark prediction showed modest internal discrimination, and the 90 min arterial-gas model was only numerically higher, with an AUROC-difference interval crossing zero. Because development and validation used the same source cohort, transportability is unproven. The Perioperative ORACLE trial also illustrates the need to evaluate clinical benefit in addition to model performance (41); external validation and assessment of calibration and clinical utility remain necessary (30).

Residual confounding and selection bias remain important limitations. Pulmonary function, diffusing capacity, tumour stage, one-lung-ventilation timing and duration, pulmonary perfusion, and physiological dead space were unavailable. Observation weighting addressed only measured predictors of gradient availability. The E-value (2.31 for the point estimate; 1.55 for the confidence limit nearest the null) quantifies sensitivity to unmeasured confounding but does not eliminate it. The *post hoc* conceptual DAG (Supplementary Figure S1) clarifies these assumptions without implying causal identification.

The 80 events also constrained model complexity. The primary model was parsimonious (10 slope parameters), and the conventional estimate, 1,000-refit bootstrap interval, and Firth estimate were closely concordant, reducing concern about substantial small-sample coefficient inflation but not eliminating uncertainty. The study was not preregistered and was reconstructed after data access. The 108 overlapping specifications were descriptive robustness analyses; Holm adjustment was limited to the three interaction tests. Secondary and exploratory findings therefore remain supportive rather than confirmatory.

Prospective studies should obtain arterial gases at defined operative phases, record one-lung ventilation timing and duration, measure dead space and pulmonary perfusion, and adjudicate postoperative respiratory outcomes. Multimodal studies could also evaluate whether preoperative biological markers, pulmonary function, and standardised intraoperative physiology provide complementary information. Higher-resolution datasets such as VitalDB may help examine physiological trajectories, although INSPIRE and VitalDB contain overlapping surgical cases from the same institution and overlap must be assessed when both are used (17, 42). Independent multicentre validation should directly compare models with and without the gradient and evaluate discrimination, calibration, and clinical net benefit before the gradient is used to guide treatment.

## Conclusion

A higher median intraoperative PaCO_2_–EtCO_2_ gradient was associated with database-derived postoperative respiratory morbidity after lung cancer resection. However, a *post hoc* incremental analysis did not demonstrate meaningful improvement in internally cross-validated risk prediction beyond PaCO_2_ and measured clinical covariates. Prospective studies with standardised arterial-gas sampling, clinically adjudicated respiratory outcomes, and independent external validation are required before the gradient can be considered for clinical risk stratification or perioperative decision-making.

## Data Availability

The dataset analysed in this study is INSPIRE version 1.4.2, available from PhysioNet at https://physionet.org/content/inspire/1.4.2/ (10.13026/1eay-yc85). Access requires PhysioNet credentialing, completion of the required training, and acceptance of the applicable data-use agreement.
