# Supplementary material for: Higher intraoperative arterial-to-end-tidal carbon dioxide gradients are associated with postoperative respiratory morbidity after lung cancer resection: a retrospective INSPIRE cohort study
Source: Front Med (Lausanne). 2026 Oct 15;13:1995794. doi: 10.3389/fmed.2026.1995794 (PMC13716742; doi:10.3389/fmed.2026.1995794)
Supplement: Supplementary file 1 [file Supplementaryfile1.docx]

**Supplementary Material**

**Higher intraoperative arterial-to-end-tidal carbon dioxide gradients are associated with postoperative respiratory morbidity after lung cancer resection: a retrospective INSPIRE cohort study**

Long Li, Dan Wang, Qingyi Zhou, Bin Shang

# Supplementary Figure S1. Conceptual framework for covariate selection

The post hoc conceptual directed acyclic graph (DAG) was developed during revision to clarify the assumptions underlying covariate selection. Blue nodes represent measured baseline or operative-context variables included in the primary adjustment set. The yellow node represents measured time-varying intraoperative factors evaluated in expanded or sensitivity models. The dashed grey node represents unavailable factors that may cause residual confounding. The red exposure–outcome arrow indicates the target association and should not be interpreted as proof of a causal effect.

Supplementary Figure S1. Post hoc conceptual DAG illustrating assumed relationships relevant to covariate selection. The figure was developed during revision to improve transparency and does not establish causal identification. Measured time-varying intraoperative factors were evaluated in expanded or sensitivity models; unavailable factors are shown as potential sources of residual confounding.

# Supplementary Methods: exploratory trajectory analyses

EtCO₂, MAP, and VT/PBW were summarised in 20 equal bins of relative surgical time, requiring at least two observed bins per channel for each patient. Missing bins were linearly interpolated, with the nearest observed value carried to each boundary. After subtracting each patient's mean EtCO₂, ordinary principal component analysis (PCA) was applied to the trajectory matrix. The first five standardised component scores were entered separately into outcome models adjusted for the primary covariates and median gradient. This fixed-grid approach was used for exploratory summarisation rather than as a formal functional-data model for sparse longitudinal measurements (1).

Gaussian hidden Markov models (HMMs) with diagonal emission covariances were used to explore data-derived patterns in EtCO₂, MAP, and VT/PBW. Channels were standardised across eligible trajectories while retaining patient sequence boundaries. For each model with two to six states, three initialisations and up to 500 expectation–maximisation iterations were used, retaining the solution with the highest likelihood. Standardised emission variances were constrained to at least 0.01 to prevent collapse at repeated discretised values. State occupancy was the proportion of 20 decoded bins assigned to a state. For the state with the lowest mean MAP, outcome associations were estimated per 10-percentage-point increase in occupancy and per additional entry; starting in the state did not count as an entry. These analyses were inspired by prior state-space and high-resolution physiological modelling work (2, 3), but the states identified here were statistical constructs and not validated clinical or physiological phenotypes.

Confidence intervals for HMM-derived associations were conditional on the learned states. HMM initialisations used the same reproducible random-seed scheme as the original analysis. The analyses were exploratory and hypothesis-generating and were not used as confirmatory evidence.

# Supplementary Results: exploratory trajectory analyses

Trajectories were constructed for 2,607 patients. The first five EtCO₂ principal components explained 27.5%, 20.9%, 10.7%, 8.0%, and 6.0% of the variance. Among 2,398 complete cases with 79 respiratory morbidity events, none of the five component scores was clearly associated with the outcome after adjustment for the median gradient and primary covariates (all nominal P≥0.145).

For HMMs with two to six states, Bayesian information criterion decreased as the number of states increased, and all retained fits converged. In the illustrative five-state model, the lowest state mean MAP was 81.0 mmHg, above conventional hypotension thresholds. Across models with different numbers of states, occupancy ORs per 10 percentage points ranged from 0.97 to 1.07 and entry associations varied. There was no consistent association between respiratory morbidity and the state with the lowest mean MAP. These findings did not provide consistent evidence beyond the patient-level median gradient.

# Supplementary Figure S2. Exploratory analyses of intraoperative trajectories

Supplementary Figure S2. Exploratory analyses of intraoperative trajectories. (A) Variance explained by the first five ordinary PCA components of mean-centred EtCO₂ trajectories in 2,607 patients. (B) Outcome associations per standard-deviation increase in each component score, adjusted for primary covariates and median gradient, in 2,398 complete cases with 79 events. (C) HMM Bayesian information criterion for two to six states. (D) Emission means in the illustrative five-state model, ordered by MAP. Numbers retain the original units: mmHg for EtCO₂ and MAP, and mL/kg for VT/PBW. (E–F) Conditional outcome associations for occupancy of, and entries into, the state with the lowest mean MAP. Occupancy effects are per 10 percentage points; entry effects are per additional transition. The HMM states are data-derived statistical constructs and should not be interpreted as validated clinical or physiological phenotypes.

# **Supplementary References**

1. Yao F, Müller HG, Wang JL. Functional Data Analysis for Sparse Longitudinal Data. J Am Stat Assoc (2005) 100:577–590. doi: 10.1198/016214504000001745

2. Contreras M, Silva B, Shickel B, Davidson A, Ozrazgat-Baslanti T, Ren Y, et al. Real-time prediction of intensive care unit patient acuity and therapy requirements using state-space modelling. Nat Commun (2025) 16:7315. doi: 10.1038/s41467-025-62121-1

3. Hyland SL, Faltys M, Hüser M, Lyu X, Gumbsch T, Esteban C, et al. Early prediction of circulatory failure in the intensive care unit using machine learning. Nat Med (2020) 26:364–373. doi: 10.1038/s41591-020-0789-4
